# An extended Tolerance Factor approach for organic–inorganic perovskites†
†Electronic supplementary information (ESI) available: SI-Table 1, shows all 2352 calculated Tolerance Factors. SI-Fig. 1–6, show plots similar to Fig. 1 for fluoride, chloride, bromide, cyanide, borohydride and azide based permutations. See DOI: 10.1039/c5sc00961h



**DOI:** 10.1039/c5sc00961h

**Published:** 2015-04-14

**Authors:** Gregor Kieslich, Shijing Sun, Anthony K. Cheetham

**Affiliations:** a Department of Materials Science and Metallurgy , University of Cambridge , 27 Charles Babbage Road , Cambridge CB3 0FS , UK . Email: gk354@cam.ac.uk ; Email: akc30@cam.ac.uk

## Abstract

Goldschmidt's concept of ionic Tolerance Factors was recently shown to be a valuable guideline for the preparation of new compounds within the field of organic–inorganic perovskites. Here, we extend this approach and calculate Tolerance Factors for over 2500 amine–metal–anion permutations of the periodic table. The results suggest the potential existence of more than 600 undiscovered hybrid perovskites including alkaline earth metal and lanthanide based materials.

## Abstract 2

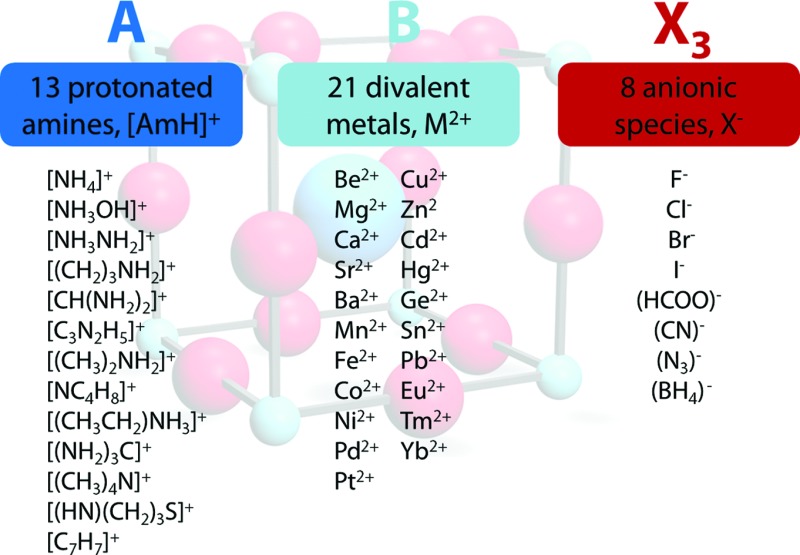
Tolerance Factors of possible hybrid perovskites are calculated for over 2500 amine-metal-anion permutations of the periodic table.

## 

The large family of solid state perovskites with general formula ABX_3_ covers a wide range of fascinating properties, which include both application-oriented phenomena and fundamental physics and chemistry.^1^ One of the key aspects that make perovskites so successful is the adaptability of this structure type towards A, B or X site substitution, which allows for tailoring of properties to meet particular requirements.^2^

During recent years a structurally related family, today known as organic–inorganic hybrid perovskites, has attracted a great deal of attention.^3,4^ In this relatively young family the perovskite architecture is essentially maintained, while at least one ion – usually A or X – is substituted by an organic ion which makes the perovskite a *hybrid*. Today, research in this emerging field is largely driven by the intriguing properties of the hybrid perovskite [CH_3_NH_3_]PbI_3_ in thin film solar cell devices.^5,6^ However, other hybrid perovskites, *e.g.* the family of perovskite-like metal formates AB(HCOO)_3_ (A = protonated amine, B = divalent metal),^7,8^ also show interesting properties such as multiferroic^9^ behaviour and tuneable mechanical properties.^10,11^

Recently, Goldschmidt's Tolerance Factor (TF) concept, which has been a central mantra in the development of solid state perovskites for decades, was extended to the emerging field of organic–inorganic perovskites.^12^ Similar to Goldschmidt's initial approach,^13^ TFs of hybrid perovskites can be obtained by treating all ions as hard spheres or cylinders in the case of certain molecular anions. The definition of an ionic radius of organic ions is challenging,^14^ but under the assumption of rotational freedom around the centre of mass and using given crystallographic data, a semi-empirical approach leads to a consistent set of effective ionic radii for organic ions.^12^ The concept of describing protonated amines in a perovskite structure as hard spheres is supported by microwave, ^14^N NMR and diffraction studies, as well as theoretical work. This reveals the highly dynamical nature of protonated amines within the perovskite cavity.^15–18^ Following this concept, TFs of lead halide, divalent metal formate, and tetrahydroborate based perovskites have been calculated and very good agreement between experimental observations and theory was found.^12,19,20^ For example, the TF concept has been successfully used as a guide in the synthesis of complex hydride perovskite-type materials, including the system [CH_3_NH_3_]Ca(BH_4_)_3_.^19^ According to the current state of knowledge, hybrid perovskites are expected to form for TFs between 0.8 and 1, as in the case of solid state perovskites. This finding also includes hybrid formates with an anti–anti formate connectivity; however, for syn–anti bridged formates, the size of the ReO_3_-like cavity is reduced and even TFs below 0.8 lead to a perovskite-like architecture.^21^

The TF concept can also help to explain the existence of low-dimensional materials with perovskite-like building motifs. The sole usage of amines that do not fit into the ReO_3_ cavity (TFs > 1) leads to low dimensional compounds, *e.g.* [NC_4_H_8_]CdCl_3_, [C_7_H_7_]PbI_3_ and [(CH_3_CH_2_)NH_3_]PbI_3_.^22–24^ In such compounds 1D chains of face-sharing octahedra are separated by protonated amines. A change in stoichiometry consequently leads to a different class of 2D materials such as [C_2_H_5_NH_3_]_2_CuCl_4_, [CH_3_NH_3_]_2_FeCl_4_ and [C_4_H_9_NH_3_]_2_MnCl_4_,^25^ where single layers of corner sharing MX_6_-octehedra are separated by large protonated amines. The partial substitution of the large amine with a smaller amine then leads to compounds such as ((C_4_H_9_)NH_3_)_2_(CH_3_NH_3_)_2_Sn_3_I_7_ and (NH_2_CNH_2_)_2_(CH_3_NH_3_)_2_Sn_2_I_8_.^26^ In these Ruddlesden–Popper type phases, perovskite-layers with TFs between 0.8 and 1.0 containing the smaller amine are separated by larger protonated amines that do not fit into the ReO_3_-like cavity.^4^

Motivated by the latest developments in this fast moving field, we have calculated Tolerance Factors for a wide range of possible amine–metal–anion permutations in the periodic table and shown that a vast number of hybrid perovskites remain to be discovered.

## Approach and discussion

**Fig. 1:**
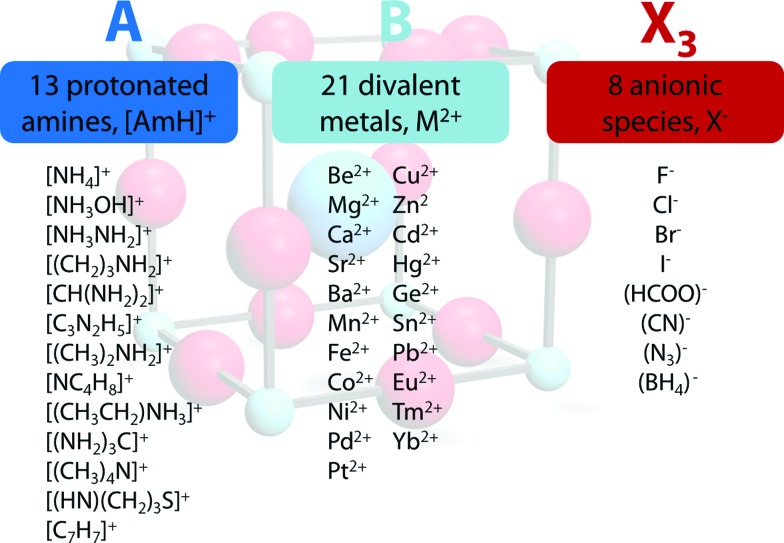
Overview of the general approach applied in this work. In all, 2352 TFs were calculated, of which 742 (562 organic anion based, 180 halide based) lie in the range 0.8 < TF < 1.

Similar to Goldschmidt's initial approach, (effective) ionic radii are used for calculating Tolerance Factors of hybrid perovskites. Protonated amines are treated as spheres with an effective radius *r*_A,eff._, and organic ions as cylinders with effective height *h*_X,eff._ and effective radius *r*_X,eff._ of the cylinder, respectively. A detailed description of how to obtain effective ionic radii of organic ions is given in ref. 12. TFs of hybrid perovskites were calculated according to Goldschmidt's equation, 
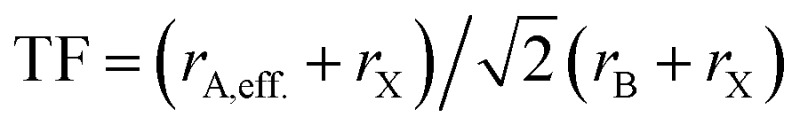
 with TF being the Tolerance Factor, *r*_A,eff._, the effective ionic radius of the protonated amine A, *r*_X_ the ionic radius of the anion X, and *r*_B_ the ionic radius of the divalent metal ion B in a perovskite with the general formula ABX_3_. TFs of the hybrids with molecular (organic) anions were calculated using the modified Goldschmidt's equation 
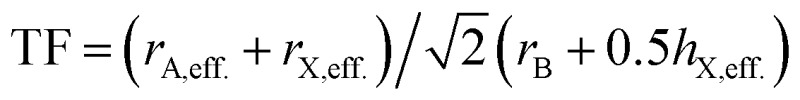
. The effective radii and heights of molecular anions were calculated according to our previous publication.^12^ The ionic radii from Shannon^14^ were used for the divalent metal cations Be^2+^, Mg^2+^, Ca^2+^, Sr^2+^, Ba^2+^, Mn^2+^, Fe^2+^, Co^2+^, Ni^2+^, Pd^2+^, Pt^2+^, Cu^2+^, Zn^2+^, Cd^2+^, Hg^2+^, Ge^2+^, Sn^2+^, Pb^2+^, Eu^2+^, Tm^2+^, Yb^2+^ and the halides, F^–^, Cl^–^, Br^–^, I^–^. For BH_4_^–^ we used a radius of 203 pm.^27^ Divalent metal cations were selected according to the stability of the divalent oxidation state in coordination compounds. The effective radii of the protonated amines [NH_4_]^+^ (ammonium, 146 pm), [NH_3_OH]^+^ (hydroxylammonium, 216 pm), [CH_3_NH_3_]^+^ (methylammonium, 217 pm), [NH_3_NH_2_]^+^ (hydrazinium, 217 pm), [(CH_2_)_3_NH_2_]^+^ (azetidinium, 250 pm), [CH(NH_2_)_2_]^+^ (formamidinium, 253 pm), [(C_3_N_2_H_5_]^+^ (imidazolium, 258 pm), [(CH_3_)_2_NH_2_]^+^ (dimethylammonium, 272 pm), [(CH_3_CH_2_)NH_3_]^+^ (ethylammonium, 274 pm), [(NH_2_)_3_C]^+^ (guanidinium, 278 pm) and [(CH_3_)_4_N]^+^ (tetramethylammonium, 292 pm) were used from ref. 12. The effective radii of [C_3_H_4_NS]^+^ (thiazolium, 320 pm) and [NC_4_H_8_]^+^ (3-pyrollinium, 272 pm) were obtained following the procedure described in previous work.^12,22,23^ For [C_7_H_7_]^+^ (tropylium), a radius of 333 pm was used which was calculated by using an ionic radius of 177 pm for C.^12,23,28^ Following our recent publication, the errors of obtained Tolerance Factors are estimated at round 6%. An overview of the applied procedure and the ions used is given in Fig. 1.

The permutations of 13 different protonated amines, 8 different anionic species and 21 divalent metal ions lead to 2352 TFs. A full list of all calculated TFs is given in the ESI (Table SI-1†). Out of 2352 calculated TFs, 742 show TFs between 0.8 and 1.0 that include approximately 140 known materials, such as the well characterized hybrid perovskites [CH_3_NH_3_]PbI_3_ (TF = 0.912), [CH_3_NH_3_]SnI_3_ (TF = 0.922), [CH_3_NH_3_]EuI_3_ (TF = 0.917), the series of [AmH]M(HCOO)_3_ (4^12^·6^3^ topology and anti–anti formate bridges) and [AmH]M(N_3_)_3_, and metastable compounds, [NH_4_]CaCl_3_ (TF = 0.823).^5,7,26,29,30^ To the best of our knowledge, only a few formate based compounds are known where TFs considerations lead to wrong conclusions, although preliminary DFT results suggest the existence of polymorphism in some of these frameworks. *e.g.* [NH_3_NH_2_]Mg(HCOO)_3_ (TF = 0.845, a 4^9^·6^6^ metal-formate framework with chiral hexagonal channels).^31^ The TF considerations discussed above predict around 600 hypothetical organic–inorganic perovskites that have not yet been reported. Clearly, it is not expected that all of the 600 hypothetical compounds can be made, nor that they will be stable under ambient conditions, but the experience to date with TFs points to the likelihood that many of these compounds will indeed be discovered. The strength of this semi-empirical approach in comparison with more accurate, modern computational techniques lies in its simplicity,^32^ making it a powerful and rapid tool for experimental scientists.

**Fig. 2:**
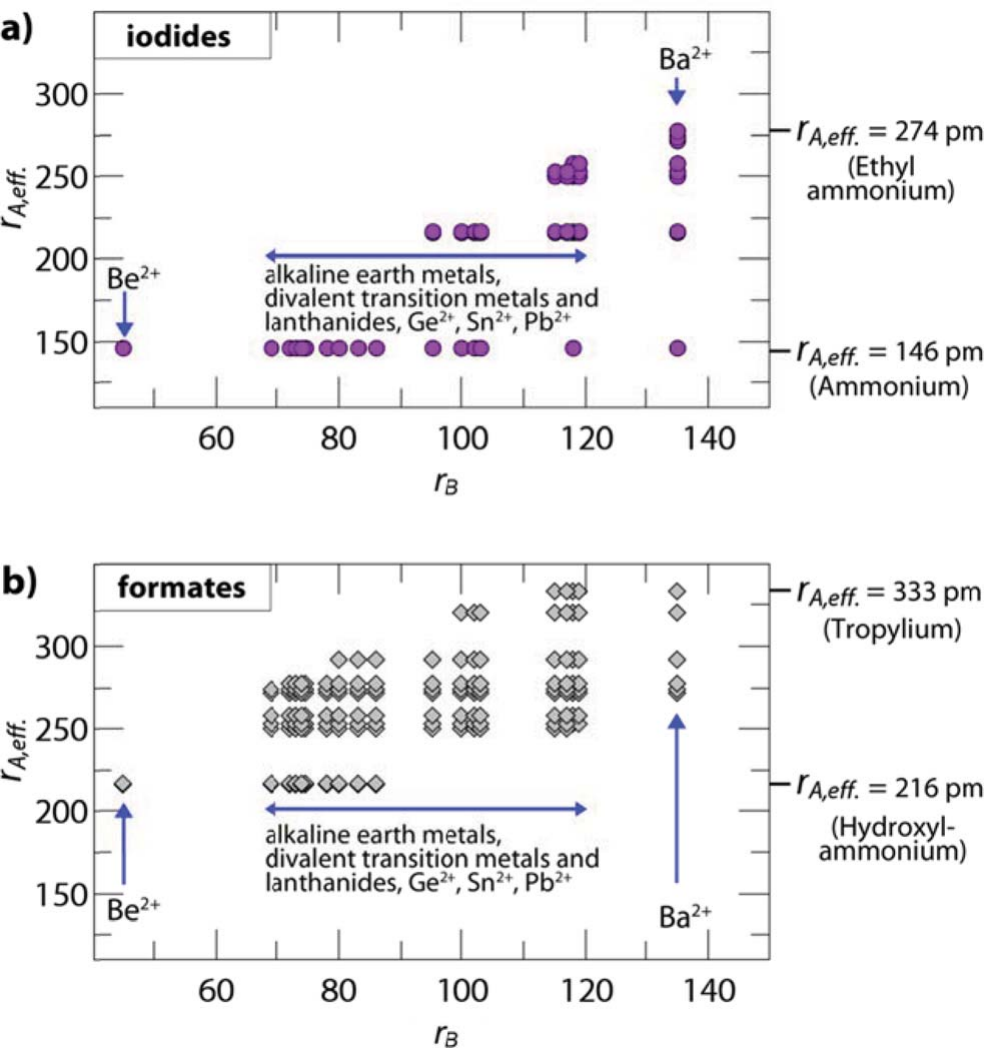
Plotted are the effective radii of protonated amines *r*_A,eff._ *vs.* ionic radii of the divalent metals *r*_B_ of hypothetical compositions. The figure shows (**a**) iodide based and (**b**) formate based permutations with TFs between 0.8 and 1.0. The plots highlight the influence of the linking anion on the size-ranges of protonated amines and divalent metals where a hybrid perovskite structure is expected to form. The anisotropy of the anti–anti bridged formate and the associated smaller effective radius (*r*_X,eff._) leads to a larger ReO_3_-like cavity, so that larger protonated amines can fit within the cavity for a fixed divalent metal. In the case of formates with a 4^12^·6^3^ topology and syn–anti connectivity, the size of the ReO_3_ cavity is reduced and even smaller protonated amines lead to a perovskite-like architecture, as recently shown for [NH_4_]Cd(HCOOH)_3_.^21^

The influence of the size and anisotropy of the linking anion on the TFs is highlighted in Fig. 2 for iodides and formates, while the S.I. contain plots for all other halides, cyanides, azides and borohydrides. Comparing iodides (*r*_X_ = 220 pm) and anti–anti formates (*r*_X,eff._ = 136 pm, *h*_X,eff._ = 447 pm), the anisotropy of the organic formate together with its smaller *r*_X,eff._ leads to a larger ReO_3_-like cavity so that amines with larger effective radii can be incorporated into the framework. At the same time, the incorporation of small protonated amines would lead to a reduced packing density (TF < 0.8). Consequently, for a fixed divalent metal the size-range of protonated amines that fit in the cavity is shifted to higher values. For example, for B = Cd^2+^ (*r*_B_ = 95 pm), TFs between 0.8 and 1.0 can be found for *r*_A,eff._ (A-CdI_3_) = 146–217 pm and *r*_A,eff._ (A-Cd(HCOO)_3_) = 250–292 pm (anti–anti connectivity of the formate).

Today, promising synthetic approaches towards hybrid perovskite frameworks include solid–solid diffusion-based reactions and solution chemistry methods. For example, a simple diffusion based solid-state reaction at 100–200 °C can be used for the preparation of CH_3_NH_3_PbI_3_ and CH_3_NH_3_EuI_3_ starting from MI_2_ (M = Pb^2+^, Eu^2+^) and CH_3_NH_3_I.^29,33^ This reaction scheme looks very comprehensive for the preparation of halide-based hybrids and could potentially be applied to most of the metal cations considered in this work. However, not all protonated amines, *e.g.* azetidinium, hydrazinium and hydroxylammonium, are stable at the required temperatures and new synthetic procedures must be developed. For molecular anion based hybrids, solution chemistry approaches provide a powerful method in many cases.^34,35^

The wide applicability of TFs for hybrid perovskites suggests that the packing density is a crucial factor for the formation of a 3D hybrid perovskite framework. Within hybrid frameworks, the complexity of the bonding allows for tuning different properties over a wide range, *e.g.* band-gap engineering due to B or X site substitution in halide based compounds or altering magnetic properties in azido-bridged frameworks.^34,36^ Hydrogen bonding interactions between the protonated amine and the cavity have been shown to have a large impact on the materials' properties.^10^ For instance, in iodide and formate based frameworks, bonding interactions between amines and the frameworks influence the mechanical properties and are responsible for temperature-driven order–disorder transitions.^15–17,32,37^

In conclusion we calculated Tolerance Factors for over 2300 hypothetical amine–metal–anion permutations based on halides and molecular (*organic*) anions such as (HCOO)^–^, (CN)^–^ and (N_3_)^–^. Of these, 742 show TFs between 0.8 and 1 are predicted to adopt the perovskite structure. To our knowledge, the group of 742 compounds includes about 140 known examples and more than 600 unknown hypothetical compounds. Interestingly, the doping of [CH_3_NH_3_]PbI_3_ on the lead site with Ca^2+^, Sr^2+^ and Cd^2+^ was recently reported and is encouraging for further research.^38^ However, most of the predicted compounds herein, *e.g.* [CH_3_NH_3_]SrI_3_ and [CH_3_NH_3_]CaI_3_, are not expected to be good electrical conductors. More likely, related to their hybrid nature, they may show ferroelectric and ferroelastic properties which are of great importance for switching devices and sensor applications. In this context, alkaline earth and first row transition metal based hybrids are of particular interest due to their high abundance.^39^ Other compounds, such as lanthanide based hybrids, are expected to show interesting optical properties. Together with the large tuning adaptability of the perovskite structure, we expect that the family of hybrid perovskites will have a prominent influence on materials science in the future.
