# Supplementary material for: An extended Tolerance Factor approach for organic–inorganic perovskites
Source: Chem Sci. 2015 Apr 14;6(6):3430–3. doi: 10.1039/c5sc00961h (PMC5492664; doi:10.1039/c5sc00961h)
Supplement: Supplementary file 1 [file SC-006-C5SC00961H-s001.pdf]

Electronic Supplementary Material (ESI) for Chemical Science.
This journal is © The Royal Society of Chemistry 2015

SUPPORTING INFORMATION

An Extended Tolerance Factor Approach for Organic-Inorganic Perovskites

Gregor Kieslich, Shijing Sun and Anthony K. Cheetham*

Department of Materials Science and Metallurgy, University of Cambridge, 27 Charles Babbage Road, Cambridge CB3 0FS, UK

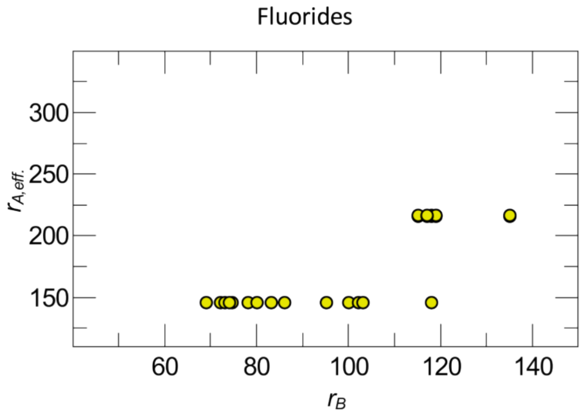

SI-Figure 1. Plotted are the effective radii of protonated amines $r_{A,eff.}$ *vs* ionic radii of the divalent metals r_B of potentially existing fluorides.

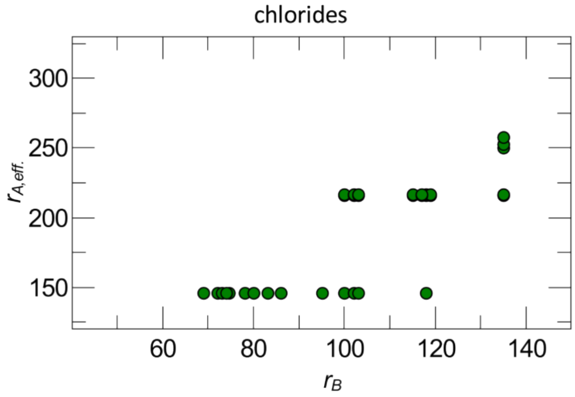

SI-Figure 2. Plotted are the effective radii of protonated amines $r_{A,eff.}$ *vs* ionic radii of the divalent metals r_B of potentially existing chlorides.

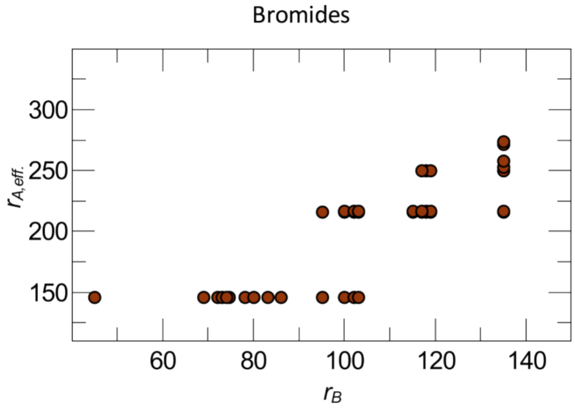

SI-Figure 3. Plotted are the effective radii of protonated amines $r_{A,eff.}$ *vs* ionic radii of the divalent metals r_B of potentially existing bromides.

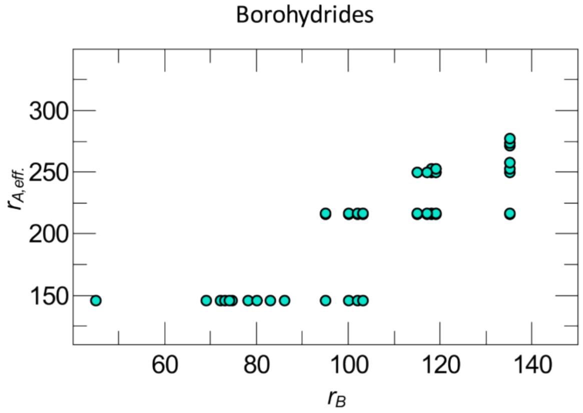

SI-Figure 4. Plotted are the effective radii of protonated amines $r_{A,eff.}$ *vs* ionic radii of the divalent metals r_B of potentially existing borohydrides.

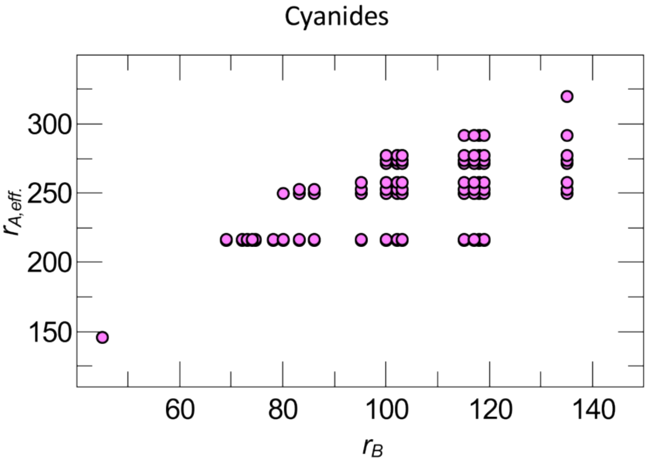

SI-Figure 5. Plotted are the effective radii of protonated amines $r_{A,eff.}$ *vs* ionic radii of the divalent metals r_B of potentially existing cyanides.

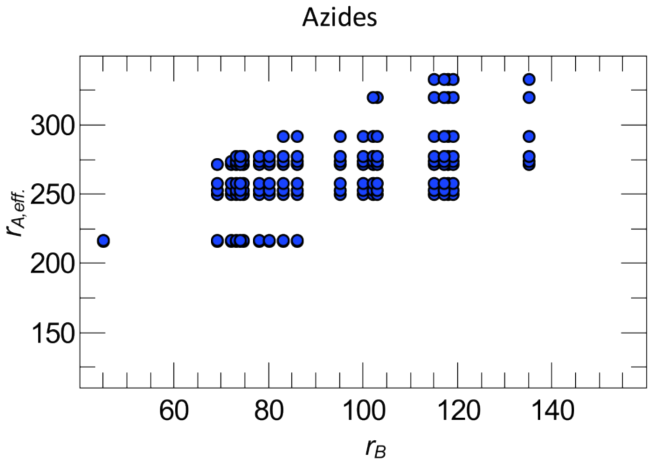

SI-Figure 6. Plotted are the effective radii of protonated amines $r_{A,eff.}$ *vs* ionic radii of the divalent metals r_B of potentially existing azides.

SI-Table 1. Shown are Tolerance Factors of all 2352 amine-metal-anion permutations regarded in this work.

**Count**	
**TFs:**	**2352.000**
**0.8 < TF < 1:**	**742.000**
**0.8 < TF < 1 (organic anions):**	**562.000**
**0.8 < TF < 1 (halides only):**	**180.000**

		**Be**	**AmH-M-F**	**AmH-M-Cl**	**AmH-M-Br**	**AmH-M-I**	**AmH-M-BH4**	**AmH-M-HCOO**	**AmH-M-CN**	**AmH-M-N3**
Ammonium	146	**45**	1.119	1.023	1.003	0.977	0.995	0.743	0.851	0.760
Hydroxylammonium	216	**45**	1.404	1.242	1.209	1.163	1.195	0.927	1.056	0.943
Methylammonium	217	**45**	1.408	1.245	1.212	1.166	1.198	0.930	1.058	0.945
Hydrazinium	217	**45**	1.408	1.245	1.212	1.166	1.198	0.930	1.058	0.945
Azetidinium	250	**45**	1.543	1.349	1.309	1.254	1.292	1.017	1.155	1.031
Formamidinium	253	**45**	1.555	1.358	1.317	1.262	1.300	1.024	1.163	1.039
Imidazolium	258	**45**	1.575	1.374	1.332	1.275	1.314	1.038	1.178	1.052
Dimethylammonium	272	**45**	1.632	1.417	1.373	1.313	1.354	1.074	1.219	1.089
Pyrrolinium	272	**45**	1.632	1.417	1.373	1.313	1.354	1.074	1.219	1.089
Ethylammonium	274	**45**	1.640	1.424	1.379	1.318	1.360	1.080	1.225	1.094
Guanidinium	278	**45**	1.657	1.436	1.391	1.329	1.371	1.090	1.236	1.104
Tetramethylammoium	292	**45**	1.714	1.480	1.432	1.366	1.411	1.127	1.277	1.141
Thiazolium	320	**45**	1.828	1.568	1.514	1.441	1.491	1.201	1.359	1.214
Tropylium	333	**45**	1.881	1.608	1.552	1.476	1.528	1.235	1.397	1.248

		**Mg**								
Ammonium	146	**72**	0.968	0.914	0.902	0.886	0.897	0.675	0.766	0.692
Hydroxylammonium	216	**72**	1.215	1.110	1.087	1.056	1.077	0.842	0.950	0.858
Methylammonium	217	**72**	1.218	1.112	1.090	1.058	1.080	0.845	0.952	0.860
Hydrazinium	217	**72**	1.218	1.112	1.090	1.058	1.080	0.845	0.952	0.860
Azetidinium	250	**72**	1.335	1.205	1.177	1.138	1.165	0.924	1.039	0.938
Formamidinium	253	**72**	1.345	1.213	1.185	1.145	1.173	0.931	1.047	0.945
Imidazolium	258	**72**	1.363	1.227	1.198	1.158	1.185	0.943	1.060	0.957
Dimethylammonium	272	**72**	1.412	1.266	1.235	1.191	1.221	0.976	1.097	0.990
Pyrrolinium	272	**72**	1.412	1.266	1.235	1.191	1.221	0.976	1.097	0.990
Ethylammonium	274	**72**	1.420	1.272	1.240	1.196	1.227	0.981	1.102	0.995
Guanidinium	278	**72**	1.434	1.283	1.251	1.206	1.237	0.991	1.112	1.004
Tetramethylammoium	292	**72**	1.483	1.322	1.288	1.240	1.273	1.024	1.149	1.038
Thiazolium	320	**72**	1.582	1.400	1.361	1.308	1.345	1.091	1.223	1.104
Tropylium	333	**72**	1.628	1.437	1.396	1.339	1.378	1.122	1.257	1.135

Ca

Ammonium	146	**100**	0.849	0.823	0.817	0.809	0.814	0.616	0.694	0.632
Hydroxylammonium	216	**100**	1.066	0.999	0.984	0.963	0.978	0.769	0.860	0.784
Methylammonium	217	**100**	1.069	1.002	0.987	0.966	0.980	0.772	0.863	0.786
Hydrazinium	217	**100**	1.069	1.002	0.987	0.966	0.980	0.772	0.863	0.786
Azetidinium	250	**100**	1.171	1.085	1.065	1.039	1.057	0.844	0.941	0.858
Formamidinium	253	**100**	1.181	1.092	1.073	1.045	1.064	0.850	0.948	0.864
Imidazolium	258	**100**	1.196	1.105	1.085	1.056	1.076	0.861	0.960	0.875
Dimethylammonium	272	**100**	1.239	1.140	1.118	1.087	1.109	0.892	0.994	0.905
Pyrrolinium	272	**100**	1.239	1.140	1.118	1.087	1.109	0.892	0.994	0.905
Ethylammonium	274	**100**	1.246	1.145	1.123	1.092	1.113	0.896	0.998	0.910
Guanidinium	278	**100**	1.258	1.155	1.132	1.100	1.123	0.905	1.008	0.918
Tetramethylammoium	292	**100**	1.301	1.190	1.166	1.131	1.155	0.936	1.041	0.949
Thiazolium	320	**100**	1.388	1.261	1.233	1.193	1.221	0.997	1.108	1.009
Tropylium	333	**100**	1.428	1.293	1.264	1.222	1.251	1.025	1.139	1.037

Sr

Ammonium	146	**118**	0.787	0.773	0.770	0.766	0.769	0.584	0.654	0.599
Hydroxylammonium	216	**118**	0.988	0.939	0.928	0.912	0.923	0.729	0.811	0.743
Methylammonium	217	**118**	0.991	0.941	0.930	0.914	0.925	0.731	0.814	0.745
Hydrazinium	217	**118**	0.991	0.941	0.930	0.914	0.925	0.731	0.814	0.745
Azetidinium	250	**118**	1.086	1.019	1.004	0.983	0.998	0.799	0.888	0.813
Formamidinium	253	**118**	1.094	1.026	1.011	0.990	1.004	0.805	0.894	0.819
Imidazolium	258	**118**	1.109	1.038	1.022	1.000	1.016	0.816	0.905	0.829
Dimethylammonium	272	**118**	1.149	1.071	1.054	1.029	1.046	0.845	0.937	0.858
Pyrrolinium	272	**118**	1.149	1.071	1.054	1.029	1.046	0.845	0.937	0.858
Ethylammonium	274	**118**	1.155	1.076	1.058	1.033	1.051	0.849	0.941	0.862
Guanidinium	278	**118**	1.166	1.085	1.067	1.042	1.060	0.857	0.950	0.870
Tetramethylammoium	292	**118**	1.206	1.119	1.099	1.071	1.090	0.886	0.982	0.899
Thiazolium	320	**118**	1.287	1.185	1.162	1.130	1.152	0.944	1.044	0.956
Tropylium	333	**118**	1.324	1.216	1.191	1.157	1.181	0.971	1.074	0.983

Ba

Ammonium	146	**135**	0.737	0.732	0.731	0.729	0.730	0.556	0.621	0.571
Hydroxylammonium	216	**135**	0.924	0.888	0.880	0.868	0.877	0.694	0.770	0.708
Methylammonium	217	**135**	0.927	0.891	0.882	0.870	0.879	0.696	0.772	0.710
Hydrazinium	217	**135**	0.927	0.891	0.882	0.870	0.879	0.696	0.772	0.710
Azetidinium	250	**135**	1.016	0.964	0.953	0.936	0.948	0.761	0.842	0.775
Formamidinium	253	**135**	1.024	0.971	0.959	0.942	0.954	0.767	0.849	0.780
Imidazolium	258	**135**	1.037	0.982	0.970	0.952	0.964	0.777	0.859	0.790

Dimethylammonium	272	**135**	1.075	1.014	1.000	0.980	0.994	0.805	0.889	0.818
Pyrrolinium	272	**135**	1.075	1.014	1.000	0.980	0.994	0.805	0.889	0.818
Ethylammonium	274	**135**	1.080	1.018	1.004	0.984	0.998	0.809	0.893	0.822
Guanidinium	278	**135**	1.091	1.027	1.013	0.992	1.006	0.817	0.902	0.829
Tetramethylammoium	292	**135**	1.128	1.058	1.043	1.020	1.036	0.844	0.931	0.857
Thiazolium	320	**135**	1.204	1.121	1.102	1.076	1.094	0.899	0.991	0.912
Tropylium	333	**135**	1.238	1.150	1.130	1.101	1.121	0.925	1.019	0.937
		**Mn**								
Ammonium	146	**83**	0.918	0.876	0.867	0.854	0.863	0.651	0.736	0.667
Hydroxylammonium	216	**83**	1.152	1.063	1.044	1.017	1.036	0.812	0.913	0.827
Methylammonium	217	**83**	1.155	1.066	1.047	1.020	1.038	0.814	0.915	0.829
Hydrazinium	217	**83**	1.155	1.066	1.047	1.020	1.038	0.814	0.915	0.829
Azetidinium	250	**83**	1.265	1.154	1.130	1.097	1.120	0.891	0.998	0.905
Formamidinium	253	**83**	1.275	1.162	1.138	1.104	1.127	0.897	1.006	0.912
Imidazolium	258	**83**	1.292	1.176	1.151	1.116	1.140	0.909	1.018	0.923
Dimethylammonium	272	**83**	1.339	1.213	1.186	1.148	1.174	0.941	1.054	0.955
Pyrrolinium	272	**83**	1.339	1.213	1.186	1.148	1.174	0.941	1.054	0.955
Ethylammonium	274	**83**	1.346	1.219	1.191	1.153	1.179	0.946	1.059	0.960
Guanidinium	278	**83**	1.359	1.229	1.201	1.162	1.189	0.955	1.069	0.969
Tetramethylammoium	292	**83**	1.406	1.267	1.237	1.195	1.224	0.987	1.104	1.001
Thiazolium	320	**83**	1.499	1.342	1.308	1.260	1.293	1.052	1.175	1.065
Tropylium	333	**83**	1.543	1.377	1.341	1.291	1.325	1.082	1.208	1.094
		**Fe**								
Ammonium	146	**78**	0.940	0.893	0.883	0.868	0.878	0.661	0.749	0.678
Hydroxylammonium	216	**78**	1.180	1.084	1.063	1.035	1.054	0.826	0.929	0.841
Methylammonium	217	**78**	1.183	1.087	1.066	1.037	1.057	0.828	0.932	0.843
Hydrazinium	217	**78**	1.183	1.087	1.066	1.037	1.057	0.828	0.932	0.843
Azetidinium	250	**78**	1.296	1.177	1.151	1.115	1.140	0.905	1.016	0.920
Formamidinium	253	**78**	1.306	1.185	1.159	1.122	1.147	0.912	1.024	0.927
Imidazolium	258	**78**	1.323	1.199	1.172	1.134	1.160	0.924	1.037	0.938
Dimethylammonium	272	**78**	1.371	1.237	1.208	1.167	1.195	0.957	1.073	0.971
Pyrrolinium	272	**78**	1.371	1.237	1.208	1.167	1.195	0.957	1.073	0.971
Ethylammonium	274	**78**	1.378	1.242	1.213	1.172	1.200	0.962	1.078	0.975
Guanidinium	278	**78**	1.392	1.253	1.223	1.182	1.210	0.971	1.088	0.985
Tetramethylammoium	292	**78**	1.440	1.291	1.259	1.215	1.246	1.004	1.124	1.017
Thiazolium	320	**78**	1.536	1.368	1.332	1.281	1.316	1.069	1.196	1.082
Tropylium	333	**78**	1.580	1.403	1.365	1.312	1.349	1.100	1.229	1.112

		**Co**								
Ammonium	146	**74.5**	0.956	0.905	0.894	0.879	0.889	0.669	0.759	0.686
Hydroxylammonium	216	**74.5**	1.200	1.099	1.077	1.047	1.068	0.835	0.941	0.850
Methylammonium	217	**74.5**	1.203	1.101	1.080	1.049	1.070	0.838	0.944	0.853
Hydrazinium	217	**74.5**	1.203	1.101	1.080	1.049	1.070	0.838	0.944	0.853
Azetidinium	250	**74.5**	1.318	1.193	1.166	1.128	1.154	0.916	1.029	0.930
Formamidinium	253	**74.5**	1.329	1.201	1.174	1.136	1.162	0.923	1.037	0.937
Imidazolium	258	**74.5**	1.346	1.215	1.187	1.148	1.175	0.935	1.050	0.949
Dimethylammonium	272	**74.5**	1.395	1.254	1.223	1.181	1.210	0.968	1.087	0.982
Pyrrolinium	272	**74.5**	1.395	1.254	1.223	1.181	1.210	0.968	1.087	0.982
Ethylammonium	274	**74.5**	1.402	1.259	1.229	1.186	1.215	0.973	1.092	0.987
Guanidinium	278	**74.5**	1.416	1.270	1.239	1.196	1.226	0.982	1.102	0.996
Tetramethylammoium	292	**74.5**	1.465	1.309	1.276	1.229	1.261	1.016	1.139	1.029
Thiazolium	320	**74.5**	1.562	1.387	1.349	1.297	1.333	1.082	1.211	1.095
Tropylium	333	**74.5**	1.608	1.423	1.383	1.328	1.366	1.113	1.245	1.125
		**Ni**								
Ammonium	146	**69**	0.983	0.925	0.913	0.896	0.907	0.682	0.775	0.699
Hydroxylammonium	216	**69**	1.233	1.123	1.099	1.067	1.089	0.851	0.960	0.866
Methylammonium	217	**69**	1.237	1.126	1.102	1.069	1.092	0.853	0.963	0.869
Hydrazinium	217	**69**	1.237	1.126	1.102	1.069	1.092	0.853	0.963	0.869
Azetidinium	250	**69**	1.355	1.219	1.190	1.150	1.178	0.933	1.051	0.948
Formamidinium	253	**69**	1.366	1.228	1.198	1.157	1.185	0.940	1.059	0.955
Imidazolium	258	**69**	1.384	1.242	1.211	1.170	1.198	0.952	1.072	0.967
Dimethylammonium	272	**69**	1.434	1.281	1.249	1.204	1.235	0.986	1.109	1.000
Pyrrolinium	272	**69**	1.434	1.281	1.249	1.204	1.235	0.986	1.109	1.000
Ethylammonium	274	**69**	1.441	1.287	1.254	1.209	1.240	0.991	1.114	1.005
Guanidinium	278	**69**	1.455	1.298	1.265	1.218	1.250	1.001	1.125	1.015
Tetramethylammoium	292	**69**	1.506	1.338	1.302	1.253	1.287	1.035	1.162	1.048
Thiazolium	320	**69**	1.606	1.417	1.377	1.321	1.360	1.102	1.236	1.115
Tropylium	333	**69**	1.652	1.454	1.412	1.353	1.393	1.134	1.271	1.146
		**Pd**								
Ammonium	146	**86**	0.905	0.866	0.858	0.846	0.854	0.644	0.728	0.661
Hydroxylammonium	216	**86**	1.136	1.051	1.033	1.008	1.025	0.804	0.903	0.819
Methylammonium	217	**86**	1.139	1.054	1.036	1.010	1.028	0.806	0.905	0.821
Hydrazinium	217	**86**	1.139	1.054	1.036	1.010	1.028	0.806	0.905	0.821
Azetidinium	250	**86**	1.248	1.141	1.118	1.086	1.108	0.882	0.988	0.896
Formamidinium	253	**86**	1.258	1.149	1.126	1.093	1.116	0.889	0.995	0.903
Imidazolium	258	**86**	1.274	1.163	1.138	1.105	1.128	0.900	1.008	0.914
Dimethylammonium	272	**86**	1.320	1.200	1.173	1.137	1.162	0.932	1.043	0.946

Pyrrolinium	272	**86**	1.320	1.200	1.173	1.137	1.162	0.932	1.043	0.946
Ethylammonium	274	**86**	1.327	1.205	1.179	1.142	1.167	0.937	1.048	0.950
Guanidinium	278	**86**	1.340	1.216	1.189	1.151	1.177	0.946	1.058	0.959
Tetramethylammoium	292	**86**	1.386	1.253	1.224	1.183	1.211	0.978	1.092	0.991
Thiazolium	320	**86**	1.478	1.327	1.294	1.248	1.280	1.042	1.162	1.054
Tropylium	333	**86**	1.521	1.361	1.326	1.278	1.311	1.072	1.195	1.084

Pt

Ammonium	146	**80**	0.931	0.886	0.876	0.863	0.872	0.657	0.744	0.674
Hydroxylammonium	216	**80**	1.168	1.076	1.056	1.028	1.047	0.820	0.922	0.835
Methylammonium	217	**80**	1.172	1.078	1.058	1.030	1.049	0.822	0.925	0.837
Hydrazinium	217	**80**	1.172	1.078	1.058	1.030	1.049	0.822	0.925	0.837
Azetidinium	250	**80**	1.284	1.168	1.143	1.108	1.132	0.899	1.009	0.914
Formamidinium	253	**80**	1.294	1.176	1.150	1.115	1.139	0.906	1.017	0.921
Imidazolium	258	**80**	1.311	1.189	1.163	1.127	1.152	0.918	1.029	0.932
Dimethylammonium	272	**80**	1.358	1.227	1.199	1.160	1.187	0.951	1.065	0.964
Pyrrolinium	272	**80**	1.358	1.227	1.199	1.160	1.187	0.951	1.065	0.964
Ethylammonium	274	**80**	1.365	1.233	1.204	1.164	1.192	0.955	1.070	0.969
Guanidinium	278	**80**	1.379	1.244	1.214	1.174	1.202	0.965	1.080	0.978
Tetramethylammoium	292	**80**	1.426	1.281	1.250	1.207	1.237	0.997	1.116	1.010
Thiazolium	320	**80**	1.521	1.357	1.322	1.273	1.307	1.062	1.187	1.075
Tropylium	333	**80**	1.565	1.393	1.355	1.303	1.339	1.093	1.221	1.105

Cu

Ammonium	146	**73**	0.963	0.910	0.899	0.883	0.894	0.673	0.763	0.689
Hydroxylammonium	216	**73**	1.209	1.105	1.083	1.052	1.073	0.839	0.946	0.855
Methylammonium	217	**73**	1.212	1.108	1.086	1.055	1.076	0.842	0.949	0.857
Hydrazinium	217	**73**	1.212	1.108	1.086	1.055	1.076	0.842	0.949	0.857
Azetidinium	250	**73**	1.328	1.200	1.172	1.134	1.161	0.921	1.035	0.935
Formamidinium	253	**73**	1.339	1.208	1.180	1.142	1.168	0.928	1.043	0.942
Imidazolium	258	**73**	1.356	1.222	1.193	1.154	1.181	0.940	1.056	0.954
Dimethylammonium	272	**73**	1.405	1.261	1.230	1.187	1.217	0.973	1.093	0.987
Pyrrolinium	272	**73**	1.405	1.261	1.230	1.187	1.217	0.973	1.093	0.987
Ethylammonium	274	**73**	1.412	1.267	1.235	1.192	1.222	0.978	1.098	0.992
Guanidinium	278	**73**	1.426	1.278	1.246	1.202	1.232	0.987	1.108	1.001
Tetramethylammoium	292	**73**	1.476	1.317	1.283	1.236	1.268	1.021	1.145	1.034
Thiazolium	320	**73**	1.574	1.395	1.356	1.303	1.340	1.087	1.218	1.100
Tropylium	333	**73**	1.620	1.431	1.391	1.335	1.373	1.118	1.252	1.131

Zn

Ammonium	146	**74**	0.959	0.907	0.896	0.880	0.891	0.670	0.760	0.687
Hydroxylammonium	216	**74**	1.203	1.101	1.079	1.049	1.070	0.837	0.943	0.852
Methylammonium	217	**74**	1.206	1.104	1.082	1.051	1.072	0.839	0.945	0.854
Hydrazinium	217	**74**	1.206	1.104	1.082	1.051	1.072	0.839	0.945	0.854
Azetidinium	250	**74**	1.322	1.195	1.168	1.130	1.156	0.917	1.031	0.932
Formamidinium	253	**74**	1.332	1.203	1.176	1.138	1.164	0.925	1.039	0.939
Imidazolium	258	**74**	1.350	1.217	1.189	1.150	1.177	0.936	1.052	0.951
Dimethylammonium	272	**74**	1.399	1.256	1.226	1.183	1.213	0.970	1.089	0.984
Pyrrolinium	272	**74**	1.399	1.256	1.226	1.183	1.213	0.970	1.089	0.984
Ethylammonium	274	**74**	1.405	1.262	1.231	1.188	1.218	0.975	1.094	0.988
Guanidinium	278	**74**	1.419	1.273	1.241	1.198	1.228	0.984	1.104	0.998
Tetramethylammoium	292	**74**	1.468	1.312	1.278	1.231	1.264	1.017	1.141	1.031
Thiazolium	320	**74**	1.566	1.389	1.351	1.299	1.335	1.084	1.214	1.097
Tropylium	333	**74**	1.612	1.425	1.385	1.330	1.368	1.115	1.248	1.127

Cd

Ammonium	146	**95**	0.868	0.838	0.831	0.822	0.828	0.626	0.706	0.642
Hydroxylammonium	216	**95**	1.090	1.017	1.001	0.979	0.994	0.781	0.875	0.796
Methylammonium	217	**95**	1.093	1.020	1.004	0.981	0.997	0.784	0.878	0.798
Hydrazinium	217	**95**	1.093	1.020	1.004	0.981	0.997	0.784	0.878	0.798
Azetidinium	250	**95**	1.197	1.104	1.084	1.055	1.075	0.857	0.957	0.871
Formamidinium	253	**95**	1.207	1.112	1.091	1.062	1.082	0.864	0.965	0.878
Imidazolium	258	**95**	1.223	1.125	1.103	1.073	1.094	0.875	0.977	0.889
Dimethylammonium	272	**95**	1.267	1.161	1.137	1.104	1.127	0.906	1.010	0.919
Pyrrolinium	272	**95**	1.267	1.161	1.137	1.104	1.127	0.906	1.010	0.919
Ethylammonium	274	**95**	1.273	1.166	1.142	1.109	1.132	0.910	1.015	0.924
Guanidinium	278	**95**	1.286	1.176	1.152	1.118	1.141	0.919	1.025	0.933
Tetramethylammoium	292	**95**	1.330	1.212	1.186	1.149	1.175	0.950	1.059	0.963
Thiazolium	320	**95**	1.419	1.284	1.254	1.212	1.241	1.012	1.127	1.025
Tropylium	333	**95**	1.460	1.317	1.285	1.241	1.272	1.041	1.158	1.054

Hg

Ammonium	146	**102**	0.842	0.817	0.812	0.804	0.809	0.613	0.689	0.629
Hydroxylammonium	216	**102**	1.057	0.992	0.978	0.957	0.971	0.765	0.855	0.779
Methylammonium	217	**102**	1.060	0.994	0.980	0.960	0.974	0.767	0.857	0.781
Hydrazinium	217	**102**	1.060	0.994	0.980	0.960	0.974	0.767	0.857	0.781
Azetidinium	250	**102**	1.161	1.077	1.058	1.032	1.050	0.839	0.935	0.852
Formamidinium	253	**102**	1.170	1.084	1.065	1.039	1.057	0.845	0.942	0.859
Imidazolium	258	**102**	1.186	1.097	1.077	1.050	1.069	0.856	0.954	0.870
Dimethylammonium	272	**102**	1.229	1.132	1.110	1.080	1.101	0.886	0.987	0.900

Pyrrolinium	272	**102**	1.229	1.132	1.110	1.080	1.101	0.886	0.987	0.900
Ethylammonium	274	**102**	1.235	1.137	1.115	1.085	1.106	0.891	0.992	0.904
Guanidinium	278	**102**	1.247	1.147	1.125	1.094	1.115	0.899	1.001	0.913
Tetramethylammoium	292	**102**	1.290	1.182	1.158	1.124	1.148	0.930	1.034	0.943
Thiazolium	320	**102**	1.376	1.252	1.224	1.186	1.213	0.991	1.100	1.003
Tropylium	333	**102**	1.416	1.284	1.255	1.214	1.243	1.019	1.131	1.031

Ge

Ammonium	146	**73**	0.963	0.910	0.899	0.883	0.894	0.673	0.763	0.689
Hydroxylammonium	216	**73**	1.209	1.105	1.083	1.052	1.073	0.839	0.946	0.855
Methylammonium	217	**73**	1.212	1.108	1.086	1.055	1.076	0.842	0.949	0.857
Hydrazinium	217	**73**	1.212	1.108	1.086	1.055	1.076	0.842	0.949	0.857
Azetidinium	250	**73**	1.328	1.200	1.172	1.134	1.161	0.921	1.035	0.935
Formamidinium	253	**73**	1.339	1.208	1.180	1.142	1.168	0.928	1.043	0.942
Imidazolium	258	**73**	1.356	1.222	1.193	1.154	1.181	0.940	1.056	0.954
Dimethylammonium	272	**73**	1.405	1.261	1.230	1.187	1.217	0.973	1.093	0.987
Pyrrolinium	272	**73**	1.405	1.261	1.230	1.187	1.217	0.973	1.093	0.987
Ethylammonium	274	**73**	1.412	1.267	1.235	1.192	1.222	0.978	1.098	0.992
Guanidinium	278	**73**	1.426	1.278	1.246	1.202	1.232	0.987	1.108	1.001
Tetramethylammoium	292	**73**	1.476	1.317	1.283	1.236	1.268	1.021	1.145	1.034
Thiazolium	320	**73**	1.574	1.395	1.356	1.303	1.340	1.087	1.218	1.100
Tropylium	333	**73**	1.620	1.431	1.391	1.335	1.373	1.118	1.252	1.131

Sn

Ammonium	146	**115**	0.797	0.781	0.778	0.773	0.776	0.589	0.661	0.605
Hydroxylammonium	216	**115**	1.000	0.948	0.937	0.920	0.932	0.735	0.819	0.750
Methylammonium	217	**115**	1.003	0.951	0.939	0.922	0.934	0.737	0.821	0.752
Hydrazinium	217	**115**	1.003	0.951	0.939	0.922	0.934	0.737	0.821	0.752
Azetidinium	250	**115**	1.099	1.030	1.014	0.992	1.007	0.806	0.896	0.820
Formamidinium	253	**115**	1.108	1.037	1.021	0.998	1.014	0.813	0.903	0.826
Imidazolium	258	**115**	1.122	1.049	1.032	1.009	1.025	0.823	0.914	0.837
Dimethylammonium	272	**115**	1.163	1.082	1.064	1.038	1.056	0.852	0.946	0.866
Pyrrolinium	272	**115**	1.163	1.082	1.064	1.038	1.056	0.852	0.946	0.866
Ethylammonium	274	**115**	1.169	1.087	1.069	1.043	1.061	0.856	0.950	0.870
Guanidinium	278	**115**	1.180	1.096	1.078	1.051	1.070	0.865	0.959	0.878
Tetramethylammoium	292	**115**	1.221	1.130	1.110	1.081	1.101	0.894	0.991	0.907
Thiazolium	320	**115**	1.302	1.197	1.173	1.140	1.163	0.953	1.054	0.965
Tropylium	333	**115**	1.340	1.228	1.203	1.167	1.192	0.980	1.084	0.992

Pb

Ammonium	146	**119**	0.784	0.771	0.768	0.763	0.766	0.582	0.652	0.598
Hydroxylammonium	216	**119**	0.984	0.936	0.925	0.909	0.920	0.727	0.809	0.741
Methylammonium	217	**119**	0.987	0.938	0.927	0.912	0.922	0.729	0.811	0.743
Hydrazinium	217	**119**	0.987	0.938	0.927	0.912	0.922	0.729	0.811	0.743
Azetidinium	250	**119**	1.081	1.016	1.001	0.980	0.995	0.797	0.885	0.810
Formamidinium	253	**119**	1.090	1.023	1.008	0.987	1.001	0.803	0.891	0.817
Imidazolium	258	**119**	1.104	1.035	1.019	0.997	1.012	0.813	0.903	0.827
Dimethylammonium	272	**119**	1.144	1.068	1.051	1.026	1.043	0.842	0.934	0.855
Pyrrolinium	272	**119**	1.144	1.068	1.051	1.026	1.043	0.842	0.934	0.855
Ethylammonium	274	**119**	1.150	1.072	1.055	1.030	1.047	0.846	0.938	0.860
Guanidinium	278	**119**	1.161	1.082	1.064	1.039	1.056	0.855	0.947	0.868
Tetramethylammoium	292	**119**	1.201	1.115	1.095	1.068	1.087	0.884	0.979	0.896
Thiazolium	320	**119**	1.281	1.181	1.158	1.126	1.148	0.941	1.041	0.954
Troplyium	333	**119**	1.319	1.212	1.187	1.153	1.177	0.968	1.070	0.980

Eu

Ammonium	146	**117**	0.791	0.776	0.773	0.768	0.771	0.586	0.657	0.601
Hydroxylammonium	216	**117**	0.992	0.942	0.931	0.915	0.926	0.731	0.814	0.745
Methylammonium	217	**117**	0.995	0.944	0.933	0.917	0.928	0.733	0.816	0.747
Hydrazinium	217	**117**	0.995	0.944	0.933	0.917	0.928	0.733	0.816	0.747
Azetidinium	250	**117**	1.090	1.023	1.008	0.986	1.001	0.802	0.890	0.815
Formamidinium	253	**117**	1.099	1.030	1.014	0.992	1.008	0.808	0.897	0.821
Imidazolium	258	**117**	1.113	1.042	1.026	1.003	1.019	0.818	0.908	0.832
Dimethylammonium	272	**117**	1.154	1.075	1.057	1.032	1.050	0.847	0.940	0.860
Pyrrolinium	272	**117**	1.154	1.075	1.057	1.032	1.050	0.847	0.940	0.860
Ethylammonium	274	**117**	1.159	1.080	1.062	1.037	1.054	0.851	0.944	0.865
Guanidinium	278	**117**	1.171	1.089	1.071	1.045	1.063	0.860	0.953	0.873
Tetramethylammoium	292	**117**	1.211	1.122	1.102	1.074	1.094	0.889	0.985	0.902
Thiazolium	320	**117**	1.292	1.189	1.166	1.133	1.156	0.947	1.048	0.959
Tropylium	333	**117**	1.329	1.220	1.195	1.160	1.184	0.974	1.077	0.986

Tm

Ammonium	146	**103**	0.838	0.814	0.809	0.801	0.806	0.611	0.687	0.627
Hydroxylammonium	216	**103**	1.052	0.988	0.974	0.954	0.968	0.762	0.852	0.777
Methylammonium	217	**103**	1.055	0.991	0.977	0.957	0.971	0.764	0.854	0.779
Hydrazinium	217	**103**	1.055	0.991	0.977	0.957	0.971	0.764	0.854	0.779
Azetidinium	250	**103**	1.156	1.073	1.055	1.029	1.047	0.836	0.932	0.850
Formamidinium	253	**103**	1.165	1.081	1.062	1.035	1.054	0.842	0.939	0.856
Imidazolium	258	**103**	1.181	1.093	1.074	1.046	1.065	0.853	0.951	0.867
Dimethylammonium	272	**103**	1.223	1.128	1.107	1.077	1.098	0.884	0.984	0.897

Pyrrolinium	272	**103**	1.223	1.128	1.107	1.077	1.098	0.884	0.984	0.897
Ethylammonium	274	**103**	1.229	1.133	1.112	1.081	1.102	0.888	0.988	0.901
Guanidinium	278	**103**	1.242	1.143	1.121	1.090	1.111	0.897	0.998	0.910
Tetramethylammoium	292	**103**	1.284	1.178	1.154	1.121	1.144	0.927	1.031	0.940
Thiazolium	320	**103**	1.370	1.247	1.220	1.182	1.209	0.988	1.097	1.000
Tropylium	333	**103**	1.410	1.280	1.251	1.211	1.239	1.016	1.127	1.028

Yb

Ammonium	146	**102**	0.842	0.817	0.812	0.804	0.809	0.613	0.689	0.629
Hydroxylammonium	216	**102**	1.057	0.992	0.978	0.957	0.971	0.765	0.855	0.779
Methylammonium	217	**102**	1.060	0.994	0.980	0.960	0.974	0.767	0.857	0.781
Hydrazinium	217	**102**	1.060	0.994	0.980	0.960	0.974	0.767	0.857	0.781
Azetidinium	250	**102**	1.161	1.077	1.058	1.032	1.050	0.839	0.935	0.852
Formamidinium	253	**102**	1.170	1.084	1.065	1.039	1.057	0.845	0.942	0.859
Imidazolium	258	**102**	1.186	1.097	1.077	1.050	1.069	0.856	0.954	0.870
Dimethylammonium	272	**102**	1.229	1.132	1.110	1.080	1.101	0.886	0.987	0.900
Pyrrolinium	272	**102**	1.229	1.132	1.110	1.080	1.101	0.886	0.987	0.900
Ethylammonium	274	**102**	1.235	1.137	1.115	1.085	1.106	0.891	0.992	0.904
Guanidinium	278	**102**	1.247	1.147	1.125	1.094	1.115	0.899	1.001	0.913
Tetramethylammoium	292	**102**	1.290	1.182	1.158	1.124	1.148	0.930	1.034	0.943
Thiazolium	320	**102**	1.376	1.252	1.224	1.186	1.213	0.991	1.100	1.003
Tropylium	333	**102**	1.416	1.284	1.255	1.214	1.243	1.019	1.131	1.031
